# Supplementary material for: Pharmacogenetics of Long-Term Outcomes of Schizophrenia Spectrum Disorders: The Functional Role of CYP2D6 and CYP2C19
Source: J Pers Med. 2023 Sep 4;13(9):1354. doi: 10.3390/jpm13091354 (PMC10532576; doi:10.3390/jpm13091354)
Supplement: Supplementary file 1 [file jpm-13-01354-s001.zip › jpm-2554037-supplementary.pdf]

Supplementary to
Unraveling the role of Pharmacogenetics in long-term outcomes of Schizophrenia Spectrum Disorders: Insights on the functional role of CYP2D6

Amrit Sandhu, et al.

Contents

	Page
**GROUP Investigators**	2
**Supplementary Methods:** Genotyping and quality control and imputation of non-genotyped variants	2
**Supplementary Table S1.** Selected SNPs for candidate gene, CYP2D6, and CYP2C19 PRSs.	3
**Supplementary Table S2.** Genotype-phenotype translation of CYP2D6	4
**Supplementary Table S3.** Genotype-phenotype translation of CYP2C19	6
**Supplementary Figure S1.** Distribution of unweighted CYP2D6 PRS,CYP2C19 PRS, and candidate gene PRS	7
**Supplementary Table S4.** Regression model outputs for core symptom trajectories.	8
**Supplementary Table S5.** Linear regression model outputs for cardiometabolic outcomes	9

GROUP Investigators:

Behrooz Z. Alizadeh[1], Therese van Amelsvoort[2], Wiepke Cahn[3,4], Lieuwe de Haan[5], Frederike Schirmbeck[5], Claudia J.P. Simons[2,6], Jim van Os[7,8] , Wim Veling[9]

1. Department of Epidemiology, University Medical Centre Groningen, University of Groningen, 9713 GZ Groningen, The Netherlands; B.Z.Alizadeh@UMCG.nl
2. Maastricht University Medical Center, Department of Psychiatry and Neuropsychology, School for Mental Health and Neuroscience, Faculty of Health, Medicine and Life Sciences, Maastricht University, 6211 LK Maastricht, The Netherlands; t.vanamelsvoort@maastrichtuniversity.nl; claudia.simons@ggze.nl
3. University Medical Center Utrecht, Department of Psychiatry, Brain Centre Rudolf Magnus, Utrecht University, 3584 CX Utrecht, Netherlands; W.Cahn@umcutrecht.nl
4. Altrecht, General Mental Health Care, 3584 CX Utrecht, the Netherlands
5. msterdam University Medical Center, University of Amsterdam, Department of Psychiatry, Amsterdam 7932, The Netherlands; l.dehaan@amsterdamumc.nl, n.f.schirmbeck@amsterdamumc.nl
6. GGzE Institute for Mental Health Care, 5611 Eindhoven, The Netherlands; claudia.simons@ggze.nl
7. University Medical Center Utrecht, Department of Translational Neuroscience, Brain Center Rudolf Mag-nus, 3584 CX Utrecht, The Netherlands; J.J.vanOs-2@umcutrecht.nl
8. King's College London, King's Health Partners, Department of Psychosis Studies, Institute of Psychiatry, 999020 London, United Kingdom; J.J.vanOs-2@umcutrecht.nl
9. Department of Psychiatry, Rob Giel Research Center, University Medical Center Groningen, University of Groningen, 9713 GZ Groningen, The Netherlands; w.veling@umcg.nl

Supplementary Methods

Genotyping and quality control and imputation of non-genotyped variants

Genotype data for 2,812 individuals (patients, siblings, parents, and healthy controls) and 570,038 SNVs was available for quality control (QC). The data was generated on a customized Illumina Institute of Psychological Medicine and Clinical Neurology array with chips containing ~250K common SNVs and 250K exome chip variants (rate, exomic, nonsynonymous, minor allele frequency (MAF) < 1%), and ~50K psychiatric-related variants. First, SNVs and samples with call rates below 95% and 98% respectively, were removed. A strict QC for a subsequent sample was then conducted. This involved removal of SNVs with a MAF threshold < 10% and those that were not in Hardy-Weinberg Equilibrium (HWE) (p-value<1e-0.5), followed by linkage disequilibrium (LD) based SNV pruning (R2<0.2) with a window size of 50, and window shifting per 5 SNVs. These steps resulted in ~58K SNVs to assess sex errors, heterozygosity (F<3 standard deviations (SD)), homozygosity (F>3SD), and relatedness by pairwise identity by descent (IBD) values. Duplicate samples were removed (identical pihat > 0.8) and remaining pairs were manually checked as the data set contains family members. These failing samples were then removed from the data set and SNVs with call rates below 98% were further removed. To check ancestry, multidimensional scaling clustering with Hapmap phase 3 was computed. Thereafter, the first 10 principal component analysis (PCA) were calculated by the EIGENSTRAT software (https://github.com/DReichLab/EIG) and individuals which deviated more than 3 standard deviations away from the Caucasian ancestry samples in the first 4 PCs were removed from the data set. Since low quality and outlier samples can affect SNV calls, these bad samples were removed and a QC was run again (SNV call rate >0.95, sample call rate >0.98, SNV call rate >0.98. SNVs with MAF<1 were not removed, as we want to identify the effects of rare variants). In total 2503 individuals and 560K SNVs passed the QC steps. After this extensive QC, pre-imputation checks were done based on the reference panel to use for imputation. SNVs were then prepared so they could be imputed on the Michigan Imputation Server (https://imputationserver.sph.umich.edu) using Minimac 4. 1000 Genome Phase 3 (version 5) reference panel (build hg19, population: Mixed) was used to phase and impute

SNVs and indels of this GROUP sample. A post imputation filter of R2>0.5 was applied to include only high quality rare variants.

Supplementary Table S1. Selected SNPs for candidate gene, CYP2D6, and CYP2C19 PRSs.

SNP	Chr	Position (HG37)	Gene	RAF	Risk allele	Alternative allele
rs6280	3	113890815	DRD3	0.320	C	T
rs6449693*	5	63256018	HTR1A	0.475	A	G
rs1062613	11	113846006	HTR3A	0.215	T	C
rs4680	22	19951271	COMT	0.477	G	A
rs16947	22	42523943	CYP2D6	0.681	G	A
rs1135840	22	42522613	CYP2D6	0.433	C	G
rs3892097	22	42524947	CYP2D6	0.203	T	C
rs1065852	22	42526694	CYP2D6	0.222	A	G
rs28371706	22	42525772	CYP2D6	0.002	A	G
rs59421388	22	42523610	CYP2D6	0.030	T	C
rs61736512	22	42525134	CYP2D6	0.030	T	C
rs1058164	22	42525132	CYP2D6	0.432	C	G
rs28371725	22	42523805	CYP2D6	0.095	T	C
rs3758581	10	96602623	CYP2C19	0.066	A	G
rs12769205	10	96535124	CYP2C19	0.142	G	A
rs4244285	10	96541616	CYP2C19	0.147	A	G
rs41291556	10	96535173	CYP2C19	0.003	C	T

*Proxy SNP with r2 = 0.99 used when the original SNP (rs6295) was not available. Rare allele frequency (RAF) was extracted from the GROUP study sample. Abbreviations: SNP, single nucleotide polymorphism; Chr, chromosome; RAF, risk allele frequency; GROUP, genetic risk for outcome of psychosis.

Supplementary Table S2. Genotype-phenotype translation of CYP2D6

Diplotypes	Number of subjects with genotype	Genotype frequency (%)	Activity Score	Predicted Phenotype
*1/*1	100	14.12	2.00	NM
*1/*2	82	11.58	2.00	NM
*1/*3	19	2.68	1.00	IM
*1/*4	109	15.40	1.00	IM
*1/*6	2	0.28	1.00	IM
*1/*9	17	2.40	1.50	NM
*1/*10	13	1.84	1.25	NM
*1/*22	2	0.28	N/A	IND
*1/*28	2	0.28	N/A	IND
*1/*33	9	1.27	2.00	NM
*1/*35	24	3.39	2.00	NM
*1/*39	1	0.14	2.00	NM
*1/*41	42	5.93	1.50	NM
*1/*131	1	0.14	N/A	IND
*2/*2	32	4.52	2.00	NM
*2/*3	3	0.42	1.00	IM
*2/*4	59	8.33	1.00	IM
*2/*6	4	0.56	1.00	IM
*2/*9	4	0.56	1.50	NM
*2/*10	4	0.56	1.25	NM
*2/*17	1	0.14	1.50	NM
*2/*22	1	0.14	N/A	IND
*2/*33	3	0.42	2.00	NM
*2/*35	8	1.13	2.00	NM
*2/*39	1	0.14	2.00	NM
*2/*41	16	2.26	1.50	NM
*2/*131	1	0.14	N/A	IND
*3/*4	5	0.71	0.00	PM
*3/*41	4	0.56	0.50	IM
*4/*4	29	4.10	0.00	PM
*4/*6	5	0.71	0.00	PM
*4/*9	7	0.99	0.50	IM
*4/*10	2	0.28	0.25	IM
*4/*28	1	0.14	N/A	IND
*4/*33	1	0.14	1.00	IM

*4/*35	15	2.12	1.00	IM
*4/*39	2	0.28	1.00	IM
*4/*41	39	5.51	0.50	IM
*4/*131	1	0.14	N/A	IND
*6/*35	2	0.28	1.00	IM
*6/*41	1	0.14	1.00	IM
*9/*10	1	0.14	0.75	IM
*9/*35	1	0.14	1.50	NM
*9/*41	3	0.42	1.00	IM
*10/33	1	0.14	1.25	NM
*10/*35	1	0.14	1.25	NM
*10/*41	4	0.56	0.75	IM
*17/*41	1	0.14	1.00	IM
*29/*35	1	0.14	1.50	NM
*33/*35	1	0.14	2.00	NM
*33/*41	1	0.14	1.50	NM
*35/*35	4	0.56	2.00	NM
*35/*41	3	0.42	1.50	NM
*35/*59	1	0.14	1.50	NM
*41/*41	3	0.42	1.00	IM
*41/*131	1	0.14	N/A	IND
Total number of subjects	701			

Note: Star alleles (*) were identified using Stargazer. Predicted phenotypes were calculated using the PharmVar translation tables (https://www.pharmvar.org/gene/CYP2C19) and Pharmacogenomics knowledge base (https://www.pharmgkb.org/page/cyp2c19RefMaterials). The two most prevalent haplotypes/ star alleles were merged to form a diplotype, allowing for identification of a metabolizer phenotype. If the metabolizer phenotype was indeterminant, no activity score was obtained, and it was denoted as N/A. This was the case for 10 individuals; these individuals with N/A activity scores were removed from subsequent analysis. Abbreviations: NM: normal metabolizers; IM: intermediate metabolizers; PM: poor metabolizers; IND: indeterminant metabolizers.

Supplementary Table S3. Genotype-phenotype translation of CYP2C19

Diplotypes	Number of subjects with genotype	Genotype Frequency (%)	Activity Score	Predicted Phenotype
*1/*1	299	42.23	2.00	NM
*1/*2	107	15.11	1.00	IM
*1/*3	1	0.14	1.00	IM
*1/*8	5	0.71	1.00	IM
*1/*17	184	25.99	2.50	RM
*2/*2	17	2.40	0.00	PM
*2/*8	1	0.14	0.00	PM
*2/*17	53	7.49	1.50	NM
*17/*17	34	4.80	3.00	UM
total number of subjects	701			

Note: Star alleles (*) were identified using Stargazer. Predicted phenotypes were calculated using the PharmVar translation tables (https://www.pharmvar.org/gene/CYP2C19) and Pharmacogenomics knowledge base (https://www.pharmgkb.org/page/cyp2c19RefMaterials). The two most prevalent haplotypes/ star alleles were merged to form a diplotype, allowing for identification of a metabolizer phenotype. Abbreviations: NM: normal metabolizers; IM: intermediate metabolizers; PM: poor metabolizers; RM: rapid metabolizers; UM: ultra-rapid metabolizers.

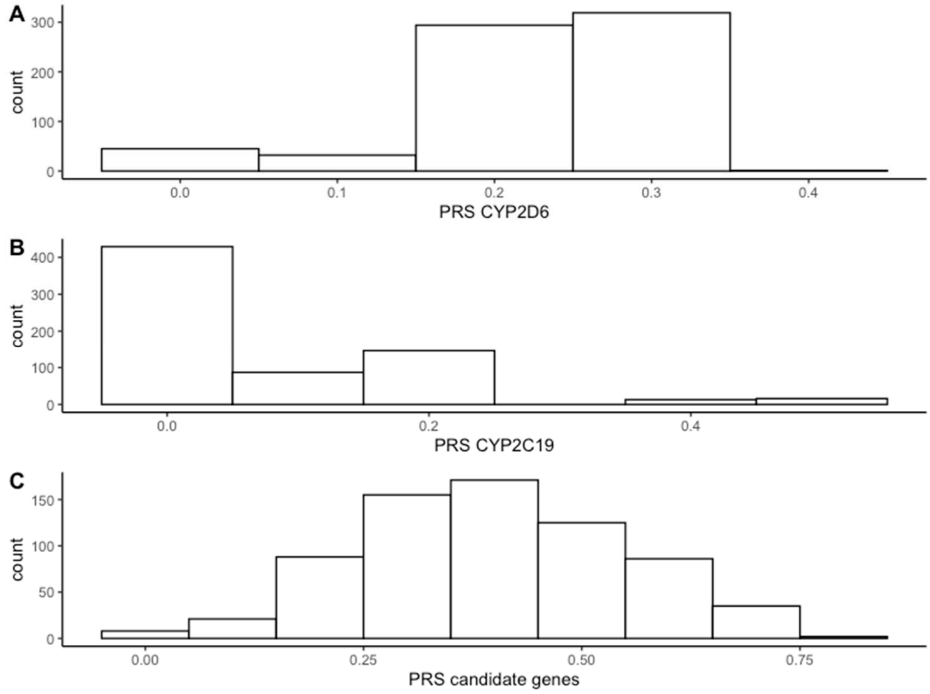

Supplementary Figure S1. Distribution of unweighted CYP2D6 PRS (A), CYP2C19 PRS (B), and candidate gene PRS (C). The x-axis reflects the PRSs and the y-axis, the number of individuals with the relative sore.

Supplementary Table S4. Regression model outputs for core symptom trajectories.

	**Pos: High**	**Neg: High**	**Cog: High**	**Cog: Mild**
**Model 1**	R^2= 0.02	R^2= 0.02	R^2= 0.02	R^2= 0.01
Sex: Male	0.65 (0.22)**	0.84 (0.23)***	0.38 (0.35)	0.30 (0.27)
Age	0.002 (0.01)	0.01 (0.01)	0.05 (0.02)**	0.02 (0.02)
**Model 2**	R^2= 0.03	R^2= 0.02	R^2= 0.03	R^2= 0.03
Sex: Male	0.52 (0.23)*	0.86 (0.23)***	0.12 (0.36)	0.13 (0.28)
Age	0.01 (0.01)	0.004 (0.01)	0.06 (0.02)**	0.02 (0.02)
Smoking	0.64 (0.20)**	-0.09 (0.18)	1.17 (0.33)***	0.73 (0.25)**
**Model 3**	R^2= 0.03	R^2= 0.02	R^2= 0.04	R^2= 0.04
Sex: Male	0.53 (0.23)*	0.86 (0.23)***	0.13 (0.36)	0.13 (0.28)
Age	0.01 (0.01)	0.004 (0.01)	0.06 (0.02)**	0.02 (0.02)
Smoking	0.64 (0.20)**	-0.09 (0.18)	1.16 (0.33)***	0.73 (0.25)**
PRS.CYP2D6:High	-0.06 (0.18)	-0.01 (0.18)	-0.14 (0.31)	-0.08 (0.25)
PRS.CYP2C19:High	-0.05 (0.18)	-0.02 (0.18)	-0.30 (0.31)	-0.17 (0.24)
PRS.cand: High	-0.23 (0.18)	-0.09 (0.18)	0.08 (0.32)	0.30 (0.26)
**Model 4**	R^2= 0.04	R^2= 0.03	R^2= 0.05	R^2= 0.05
Sex: Male	0.47 (0.23)*	0.83 (0.23)***	0.03 (0.37)	0.11 (0.28)
Age	0.01 (0.01)	0.004 (0.01)	0.06 (0.02)**	0.02 (0.02)
Smoking	0.64 (0.20)**	-0.10 (0.19)	1.18 (0.33)***	0.73 (0.25)**
CYP2D6 AS	0.38 (0.15)*	0.18 (0.14)	0.93 (0.26)***	0.20 (0.19)
CYP2C19 AS	-0.08 (0.14)	-0.05 (0.14)	0.25 (0.26)	-0.14 (0.19)

Note: *p<0.05, **p<0.01, ***p<0.001. Betas (standard errors) are displayed. Logistic regression for high positive and negative symptom trajectories references to low symptom trajectories. Multinomial logistic regression for cognitive high and mild symptom trajectories references with no impairment trajectories. McFadden's R squared calculated for positive and negative trajectories, Adjusted R-squared are displayed for cognitive symptom trajectories Abbreviations: Pos, positive symptom trajectories; Neg, negative symptom trajectories; Cog, cognitive symptom trajectories.

Supplementary Table S5. Linear regression model outputs for cardiometabolic outcomes

	**HbA1c**	**HDL**	**LDL**	**DBP**	**SBP**	**PR**	**TAG**	**BMI**	**WC**
**Model 1**	R^2= 0.07	R^2= 0.07	R^2= 0.03	R^2= 0.03	R^2= 0.05	R^2= 0.01	R^2= 0.03	R^2= 0.03	R^2= 0.05
Sex: Male	1.82 (0.77)*	-0.09 (0.02)***	0.19 (0.12)	1.99 (1.27)	8.32 (1.73)***	-1.80 (1.79)	0.30 (0.08)***	-0.53 (0.52)	4.31 (1.56)**
Age	0.25 (0.05)***	-0.002 (0.00)*	0.03 (0.01)***	0.25 (0.07)***	0.24 (0.10)*	-0.25 (0.10)*	0.01 (0.01)	0.07 (0.03)*	0.39 (0.09)***
**Model 2**	R^2= 0.07	R^2= 0.08	R^2= 0.04	R^2= 0.02	R^2= 0.05	R^2= 0.02	R^2= 0.03	R^2= 0.03	R^2= 0.05
Sex: Male	1.89 (0.78)*	-0.09 (0.02)***	0.16 (0.12)	2.03 (1.29)	8.40 (1.76)***	-2.57 (1.81)	0.30 (0.08)***	-0.55 (0.53)	4.12 (1.59)*
Age	0.25 (0.05)***	-0.003 (0.00)*	0.03 (0.01)***	0.25 (0.07)***	0.24 (0.10)*	-0.24 (0.10)*	0.01 (0.01)	0.07 (0.03)*	0.39 (0.09)***
Smoking	-0.09 (0.64)	-0.03 (0.02)	0.15 (0.10)	-0.17 (1.12)	-0.40 (1.52)	3.51 (1.56)*	0.04 (0.07)	0.10 (0.46)	0.88 (1.38)
**Model 3**	R^2= 0.07	R^2= 0.08	R^2= 0.03	R^2= 0.02	R^2= 0.05	R^2= 0.02	R^2= 0.03	R^2= 0.02	R^2= 0.05
Sex: Male	1.83 (0.79)*	-0.09 (0.02)***	0.16 (0.12)	2.10 (1.30)	8.43 (1.77)***	-2.58 (1.82)	0.30 (0.08)***	-0.52 (0.53)	4.19 (1.60)**
Age	0.25 (0.05)***	-0.002 (0.00)*	0.03 (0.01)***	0.25 (0.07)***	0.24 (0.10)*	-0.23 (0.10)*	0.01 (0.01)	0.08 (0.03)*	0.39 (0.09)***
Smoking	-0.16 (0.65)	-0.03 (0.02)	0.15 (0.10)	-0.15 (1.12)	-0.43 (1.53)	3.33 (1.57)*	0.03 (0.07)	0.11 (0.46)	0.87 (1.39)
PRS.CYP2D6:High	-0.64 (0.63)	0.0003 (0.02)	0.01 (0.1)	0.60 (1.09)	-0.11 (1.48)	-2.02 (1.53)	-0.02 (0.06)	0.25 (0.45)	0.25 (1.36)
PRS.CYP2C19:High	0.09 (0.64)	-0.01 (0.02)	-0.01 (0.10)	-0.97 (1.09)	-0.48 (1.48)	-0.054 (1.52)	-0.004 (0.07)	-0.503 (0.45)	-0.97 (1.36)
PRS.cand: High	0.06 (0.64)	0.01 (0.02)	0.004 (0.10)	-0.41 (1.10)	-0.84 (1.50)	1.12 (1.54)	0.11 (0.07)	-0.20 (0.45)	-0.66 (1.37)
**Model 4**	R^2= 0.07	R^2= 0.07	R^2= 0.03	R^2= 0.01	R^2= 0.05	R^2= 0.02	R^2= 0.023	R^2= 0.03	R^2= 0.05
Sex: Male	1.95 (0.79)*	-0.09 (0.02)***	0.16 (0.12)	1.90 (1.31)	8.28 (1.77)***	-2.7 (1.82)	0.29 (0.08)***	-0.56 (0.54)	4.08 (1.61)*
Age	0.25 (0.05)***	-0.003 (0.00)*	0.03 (0.01)***	0.25 (0.08)***	0.24 (0.10)*	-0.22 (0.10)*	0.01 (0.01)	0.08 (0.03)*	0.39 (0.09)***
Smoking	-0.08 (0.64)	-0.03 (0.02)	0.15 (0.10)	-0.06 (1.14)	-0.43 (1.52)	3.44 (1.56)*	0.03 (0.07)	0.01 (0.46)	0.86 (1.39)
CYP2D6 AS	-0.52 (0.50)	0.012 (0.01)	-0.03 (0.08)	0.80 (0.86)	0.74 (1.17)	1.18 (1.20)	0.04 (0.05)	0.02 (0.35)	0.21 (1.06)
CYP2C19 AS	-0.08 (0.49)	0.01 (0.01)	-0.01 (0.08)	0.31 (0.83)	0.004 (1.12)	1.51 (1.15)	0.02 (0.05)	0.34 (0.34)	0.48 (1.03)
	N=372	N=388	N=381	N=456	N=456	N=455	N=387	N=471	N=444

Note: *p<0.05, **p<0.01, ***p<0.001. Betas (standard errors) are displayed. High PRS scores are modelled against low PRSs. Abbreviations: PRS, unweighted polygenic risk score; AS, activity score; PRS.cand, candidate gene PRS; Glycated hemoglobin levels, HbA1c (mmol/mol); low density lipoprotein, LDL (mmol/l); high density lipoprotein, HDL (mmol/l); diastolic blood pressure, DBP (mmHg); systolic BP, SBP (mmHg); pulse rate, PR (beats/min); triglycerides, TAG (mmol/l); body mass index, BMI (kg/m2); umbilical waist circumference, WC (cm).
